# The Effect of the Economic Crisis on Adolescents’ Perceived Health and Risk Behaviors: A Multilevel Analysis

**DOI:** 10.3390/ijerph17020643

**Published:** 2020-01-19

**Authors:** Néboa Zozaya, Laura Vallejo

**Affiliations:** 1 Department of Quantitative Methods in Economics and Management, Universidad de Las Palmas de Gran Canaria, Calle Saulo Torón, 4 Las Palmas de Gran Canaria, 35017 Las Palmas, Spain; 2 Weber Economía y Salud, Calle Moreto 17, 28014 Madrid, Spain

**Keywords:** HBSC, economic crisis, recession, health, lifestyles, risk behaviors, children, teenagers, adolescents, multilevel analysis

## Abstract

Background: Previous studies have analyzed the impact of economic crises on adult’s health and lifestyles, but evidence among children and adolescents is limited. The objective of this study was to analyze the impact of the economic crisis on self-perceived health and some risk behaviors in the Spanish adolescent population. Methods: We used data from four waves (2002, 2006, 2010, 2014) of the Health Behavior in School-Aged Children (HBSC) survey in Spain. Separate multilevel logistic and linear regression models were applied for health complaints, self-rated health, life satisfaction, smoking, alcohol consumption, and breakfast skipping. Annual change in Spanish regional unemployment rates was used as a proxy of the economic crisis. An increasing set of control variables were included, consisting of individual, socioeconomic, and family and peer relationships indicators. Median odds ratios were estimated to quantify the cross-region and cross-school variation. Results: Increases in unemployment rates were linked to a higher risk of poorer health and bad habits in the simplest models. The effect was no longer statistically significant when indicators of family and peer relationships were included, suggesting a protective effect against the impact of the economic crisis. Our findings also show that schools had a larger effect on health and lifestyles than regions. Conclusion: The child’s social context—family, peers, school, and region—play an important role on the effects of the economic crisis on health and risk behaviors.

## 1. Introduction

The global economic recession initiated at the end of the first decade of the 21st century had important macroeconomic effects in most developed countries. Spain was no exception, with a gross domestic product (GDP) fall of almost 9% between 2008 and 2013, leaving high levels of debt and public deficit. The effects of the crisis quickly shifted to the level of employment. The unemployment rate of the general population rose from 7.9% in 2007 to 26.9% in 2013, with marked differences between regions, and the number of unemployed individuals increased by 4.5 million [1].

There is an extensive body of literature focused on analyzing the impact of the economic crisis on the health and lifestyles of the population, with mixed findings [1,2,3]. Evidence suggests that the crisis had a greater effect on the mental health than on the physical health of the population [1,2]. Most studies are based on analyzing the impact on the health of adults, but there is less work focused on children or adolescents.

There is a virtuous circle between good childhood health and present and future well-being. Health in childhood and youth can mark future personal, social, and academic development, and difficulties in adolescence can have important consequences in adult life [4,5]. For its part, health risk behaviors include unhealthy lifestyle habits related to nutrition, lack of physical exercise, and consumption of harmful substances (alcohol, tobacco, drugs), which can negatively affect pupils’ school performance and health [6,7].

The family is one of the most important determinants of children’s development [8]. In particular, family socio-demographic conditions have a relevant impact on the health and risk behaviors of children and adolescents. The educational level of the parents is related to adolescent lifestyles [9]. Children of parents with lower educational levels have a higher risk of poor school performance and of reporting lower life satisfaction and self-perceived health than children of parents with a university degree [10]. Family wealth also plays a crucial role in the well-being of adolescents and is a potential source of health inequalities. Adolescents from more affluent families show greater satisfaction with their lives than those from less affluent families and feel healthier than those who report that their home has few resources [1,11].

The impact of the crisis on health may differ between adults and children. Adolescents spend much of their day in school. Their environment can play an important role in their well-being. Relationships with peers and their families may affect their mental health [12]. Parental communication is one of the key ways in which the family can act as a protective health asset, helping young people to deal with stressful situations or adverse influences. Ease of parental communication and parental support are associated with positive body image, higher self-rated health, higher life satisfaction, and fewer physical and psychological complaints [13,14,15,16]. They are also less likely to participate in aggressive behaviors and substance use [17]. Schools may also have a significant effect on risk behaviors and mental health, both directly through school rules, peer influences, social activities, teacher support, and school connectedness, and indirectly by influencing student-level skills and knowledge [18,19,20].

The effects of unemployment on health and lifestyles can occur at both the individual and contextual levels [21]. For children, adult unemployment may have a dual effect. On the negative side, the fact that one or both parents lose their jobs or suffer the impact of the recession may psychologically affect the child (more stress, fear, worry) and significantly reduce family wealth [16,22,23]. On the other hand, children could also benefit, at least in the short term, from their parents’ unemployment situation by being able to spend more time with them, therefore being more available to communicate, help, cook, or control bad habits and influences [3,24]. Previous research on parental employment and youths’ well-being also suggests that parents’ unemployment is associated with young people’s lower well-being [8,16,25,26].

Parental unemployment has been found to have unintended consequences on the probability of having bad habits, such as drugs consumption, binge drinking, or smoking. Having an unemployed father has been associated with a positive effect on the probability of binge drinking [27]. The ‘economic stress’ mechanism links substance consumption to psychological reasons [28]. On the contrary, other studies indicate a positive relationship between unhealthy lifestyles and household budget, suggesting a procyclical relationship between macro-economic conditions and risk behaviors [29,30,31].

In this context, it is worth exploring the effect of the economic crisis experienced in Spain on children and adolescents. We focus on analyzing the impact on health indicators as well as on lifestyles factors, as the latter might be a mechanism that explains changes in health that have accrued and/or will develop in the long run. The aim of this work was thus to analyze the possible impact of the recession on self-perceived health and some risk behaviors of the adolescent population in Spain, taking into account family socioeconomic variables and contextual factors, and controlling for the school and regional environments.

## 2. Materials and Methods

### 2.1. Study Design

Data were obtained from the Health Behavior in School-Aged Children (HBSC), an international cross-sectional survey supported by the World Health Organization (WHO) aimed at understanding young people’s health-related behaviors, well-being, and developmental contexts [32]. The responses were collected by means of standardized self-completed questionnaires, administered in school classrooms according to standard instructions [33]. We used data from four consecutive HBSC waves (2002, 2006, 2010, and 2014) performed for Spain. Microdata were provided by the Spanish Ministry of Health, Consumption, and Social Welfare [34].

### 2.2. Sample

The sample comprised 77,651 students aged between 9 and 21 years. 0.47% of the sample (*n* = 364) were adolescents older than 18 years old, who were still enrolled in secondary education because they repeated one or more school years during their life. The mean age of the sample was 14.35 years (standard deviation (SD): ±2.22) with a balanced representation of boys and girls (49.18% boys and 50.82% girls). The students were enrolled at a total of 1181 educational centers from the 17 Spanish Autonomous Communities (plus two Autonomous Cities). Due to missing values of some of the included variables, the final sample sizes of the conducted models ranged from 53,543 to 56,507.

### 2.3. Data

There are two sets of dependent variables, regarding health and lifestyles. Adolescents’ health was measured in three alternative ways, by asking him/her about health complaints, self-rated health status, and life satisfaction (Table 1).

- Physical and psychological health complaints were measured using the HBSC symptom checklist. Pupils were asked how often in the past 6 months they had experienced somatic (headache, abdominal pain, backache, feeling dizzy) or psychological (feeling low, feeling irritable or bad-tempered, feeling nervous, and having difficulties in getting to sleep) symptoms. The response options for each item ranged from ‘about every day’ to ‘rarely or never’ (5 response options). A composite dichotomous index was created including these eight symptoms, coded as 1 when the child had rarely or never experienced any of the symptoms, and 0 otherwise. Additionally, two separate composite indexes were created for the four physical and psychological complains, following an analogue structure.
- The HBSC survey includes a question about the child’s self-perceived health, with four possible answers: excellent, good, fair, or poor. We recoded the variable into a dichotomous one, 1 being excellent or good health, and 0 fair or poor health.
- The survey also includes a 10-point visual analogue life satisfaction scale, where the child was asked to indicate the step of the ladder at which he/she would place his/her life at present (from 0 to 10). The top of the ladder indicates the best possible life and the bottom, the worst.

Three risk behaviors were also analyzed: smoking, frequent alcohol consumption, and absence of breakfast on weekdays. The variables were included individually in a dichotomous form, 1 being the bad habit and 0 the absence of it.

Of all the possible macroeconomic measures, we used unemployment as proxy of the economic crisis, as it is the most widely available indicator of economic difficulty, and previous research has shown that fluctuations in employment are more closely associated with short-term changes in health than are other economic indicators [35,36]. Poor and vulnerable members of the population are most sensitive to unemployment, and could be missed by GDP measures. We used as proxy the annual relative changes in the regional unemployment rates for the four years included in the analysis. We also checked the robustness of our findings using the change in unemployment regional rates measured in absolute percentage points. The data were extracted from the Spanish National Institute of Statistics [37].

We included several control variables in the models. Besides individual measures such as age and gender, specific socioeconomic variables were employed, as well as variables about parental relationships and conflicts with peers. Socioeconomic measures include parents’ working status (both parents are working), family structure (single versus two-parent households), and family material wealth. The family material wealth was assessed using the Family Affluence Scale (FAS) [33]. A global score was calculated as the sum of the following individual item scores [38,39]: car ownership (No: 0 points; Yes, one: 1 point; Yes, two or more: 2), having one’s own bedroom (No: 0 points; Yes: 1 point), number of computers/laptops at home (None: 0 points; One: 1 point; Two: 2 points; More than two: 3 points), and number of family holidays during the last year (None: 0 points; One: 1 point; Two: 2 points; More than two: 3 points). Using an additive score, the responses were recoded into three groups: low (0–2 points), medium (3–5 points), and high (6–9 points) family-wealth. Several indicators were also included in order to approximate family and peer relationships. Four variables aimed to measure if the child felt understood, supported, beloved, and/or known by his/her parents. Regarding peers, three variables aimed to detect bullying (give or receive) or physical fights.

### 2.4. Statistical Analysis

Multilevel regression models were applied, as data were organized at more than one level: individual, school, and region. These models were used to separately estimate the variance between pupils within the same school and region, and the variance between schools and regions. Separated models were performed for the three health outcome variables (symptoms, self-rated health status, and life satisfaction scale) and the three risk behavior variables (tobacco, alcohol consumption, and absence of breakfast), in order to assess the impact of the economic crisis on each of them.

Due to the nature of the dependent variables, logistic multilevel regression models were fitted, except for the life satisfaction scale, where linear models were used. The fixed parts of the models are a linear function of individual- and contextual-level determinants. The random parts included three variance components between regions (level 3), schools (level 2), and students (level 1).

For each dependent variable, three models were fitted by stepwise regression. Model 1 assessed the association between the outcome variable and individual-level variables (gender, age group) as well as the crisis proxy, to analyze the region- and school-level variance. Model 2 added socioeconomic variables (family wealth, parental work, and family structure) and Model 3 added family and peer relationship indicators (parental relationship and peer conflicts).

For the multilevel linear regressions on the vital satisfaction scale, residual intraclass correlation coefficients (ICC) were estimated. To quantify the cross-region and cross-school variation on health outcomes and risk behaviors of the multilevel logistic regressions, we calculated the median odds ratios (MOR) [40]. The MOR quantifies the variation between clusters by comparing two persons from two randomly chosen, different clusters. It allows us to compare between two identical individuals (level 1) that belong to different groups (region and school). In our models, it shows the extent to which the individual probability of declaring good health or a risk behavior is determined by the region where the child lives or the school of attendance.

In all models, whether the differences were significant was assessed by using the Wald chi-squared test for each predictor. The analyses were performed using the Stata 14.2 program (StataCorp., College Station, TX, USA).

## 3. Results

### 3.1. Descriptive Statistics

Table 1 and Table 2 provide a summary of the descriptive statistics. Almost half of the sample were boys (49.18%) and reported a high family affluence according to the FAS composite index (49.76%). The prevalence of several socioeconomic and relationship/conflict indicators worsened in 2010: both parents working (68.9% in 2006 versus 66.8% in 2010), biparental familiar structure (82.8% versus 79.14%), physical fights with other children (31.5% versus 33.2%), and parental knowledge (47.4% versus 46.1%) and love (87.6% versus 86.1%).

The relative change in the annual regional unemployment rates showed a different pattern over the analyzed years. The regional unemployment rates fell in relative terms by an average of 7.48% between 2005 and 2006 and 7.43% between 2013 and 2014. However, they increased by 12.07% in 2010, with substantial differences among regions. During this year of economic crisis, the unemployment rate increased in all but one region, in a range of between 23.2% and −5.7%.

Raw data on life satisfaction shows that it remained at mean levels of 6.87 by 2006 and 2010, improving up to 7.7 points by 2014. Regarding health status, 91.93% of the students reported an excellent or good health status in 2010 (3.56 percentage points more than in 2002). The proportion of students reporting good or very good health (no or few physical or psychological symptoms) dropped from 62.31% in 2006 to 61.41% in 2010 (Table 3).

Regarding lifestyles, the prevalence of regular smoking increased slightly between 2006 and 2010, with a deep decrease in 2014. Breakfast during weekdays also showed a deterioration during 2010. By contrast, the prevalence of frequent alcohol consumption decreased two percentage points between 2006 and 2010, and more than halved by 2014.

### 3.2. Multilevel Analysis

In accordance with the multilevel logistic regression models, girls presented a higher risk of reporting health complaints or a poorer health status compared with boys. Students aged 17 and older showed a higher risk of poor health than younger peers. Individuals living in more affluent households had a significantly higher probability of reporting better health (less health complaints) than those living in more deprived households. Belonging to a two-parent family structure is also related with better health outcomes. However, the fact that both parents have a job seems to have a slight positive effect on vital satisfaction and psychological health, but not on physical health or self-reported global health. The same results were observed when the symptoms were analyzed separately by dividing them into physical and psychological (Supplementary Table S1).

The four variables regarding parental support showed a positive correlation with better health and vital satisfaction (especially parental knowledge, with odds ratios between 0.36 and 1.59). By contrast, being involved in fights and bullying other peers were linked to a lower probability of enjoying good health. Being a victim of bullying by peers was a strong and significant risk factor for worse health and vital satisfaction (Table 4 and Table 5).

The changes in regional unemployment were linked to a worse health status at Models 1 and 2. However, the economic crisis proxy was not statistically significant when incorporated in the most complex models that included family and peers relationships indicators (Model 3). The effect only remained significant at a 10% significance level for vital satisfaction. Conclusions of the analyses were robust when the change in unemployment regional rates was measured in absolute percentage points.

In the multilevel models on lifestyles, the effect of gender indicates that girls are more likely to smoke and skip breakfast, but are less likely to consume alcohol than boys. Being 17 and older is associated with a higher probability for each of the risk behaviors considered. Family affluence decreases the risk of smoking and missing breakfast but does not have an effect on alcohol consumption (Model 2). The variables related with a better communication with parents (Model 3) were significantly associated with good lifestyles. By contrast, the variables regarding fights or bullying peers presented a higher risk of following these three unhealthy habits. The effect of the economic crisis remained statistically significant in all models for smoking and absence of breakfast, suggesting a positive association between increases in unemployment rate and higher risk of that bad habit. However, the size of the effect was considerably reduced when controlling for family and peer relationship indicators. The effect on alcohol consumption was no longer significant when such covariates were added.

Findings of random intercepts suggest that schools had a higher effect on health and lifestyles than regions. After adjusting for family and peer relationship indicators (Model 3), all intraclass school and region correlation coefficients (ICC and MOR) were slightly reduced in the regressions on health outcomes and smoking. MORs indicate that the school level explained between 25% and 38% of the total variance for health symptoms. School-level variance was larger for frequent alcohol consumption (MOR of 2.24–2.35) than for the other dependent variables, suggesting that the school environment has the largest effect on alcohol consumption.

## 4. Discussion

According to our results, it seems that the economic crisis is inversely related to good health and life satisfaction when controlling for individual and socioeconomic variables. However, the link between higher regional unemployment and poorer health disappears when family and peer relationships indicators are considered, suggesting a protective effect against the negative impact of the economic crisis. A similar result was found for frequent alcohol consumption. The observed detrimental effect of the economic crisis on life satisfaction, smoking, and absence of breakfast remained, but was substantially reduced when controlling for these indicators. A possible explanation for this pattern could be that tobacco and food consumption are more linked to the family income than alcohol, which is an unhealthy lifestyle that could depend to a larger extent on friends and the school context. However, this result should be considered with caution, since we were only able to analyze this alcohol variable, but not others like binge drinking.

The second relevant finding is that the school environment has an influence on health outcomes and lifestyles, especially on alcohol consumption. We found that intraclass correlation decreased for health outcomes and smoking after controlling for family and peer relationship indicators, suggesting that at least some of the identified region and school-level variance is due to parental and peer influences.

The present study contributes to the existing literature by expanding the research on the protective effect of the family and good relationships with other children against the economic crisis than hindered self-reported health and healthy lifestyles among children and adolescents. The HBSC survey has been widely used to analyze the effect of socioeconomic determinants on self-perceived health and well-being among young people [22,29,33,41,42,43]. However, to our knowledge, this is the first time that it was used to measure the potential impact of the economic crisis on health and lifestyles, jointly considering the different environment influence levels: individuals, families, schools, and regions.

Previous studies on the detrimental health effects of an economic recession on teenagers highlighted a complex causal chain between economic, social, and individual relationships [44]. Young people’s health and well-being decline was found to be anchored to parents’ unemployment [8,22,23]. The effects of both paternal and maternal job losses on child health have been associated with declines in child health in the short-run. Paternal job loss was associated with increases in depression and anxiety, while maternal job loss reduced the incidence of infectious illnesses [24]. At a macroeconomic level, higher rates of precarious employment in a region have a negative effect on people’s mental health, and likewise, lower health spending per capita [45]. However, according to other studies, the negative shift of the recent recession on the employment market has not affected adolescents’ psychological health complaints [44].

Social protection and cultural importance of families might protect adolescents from economic downturns [44]. Previous studies have demonstrated the existence of environmental influences (family, school, and friends) on the subjective health and mental health of adolescents [42,46,47,48]. Parents, teachers, and family were sources of support most consistently found to be protective against depression in children and adolescents, whereas findings were less consistent for support from friends [12]. They have been presented as a protective factor of adolescents’ well-being against adversity caused by parental unemployment [49]. Other protective factors are family autonomy and control, family and school sense of belonging, and social support at home and school [50]. School is an ideal place to improve the health and well-being of today’s children and tomorrow’s adults, but it can also be a source of anxiety and bad behaviors. In line with other studies, we have shown that health risk behaviors have relatively higher school-level variance compared to other health outcomes, although the causal mechanisms are difficult to be established [18,19]. Multilevel analyses performed in Wales using the HBSC survey have revealed that family relationships also protect from harmful substance use [42,51].

Our findings should be interpreted with caution because of several methodological limitations. The use of self-reported data on health status and socioeconomic welfare may be affected by the adolescents’ subjective perception. The cross-sectional design of the study limits causal inference. Although we used four consecutive waves of the same survey, with disaggregated microdata, no temporal follow-up of the same children could be done. Other limitation is related to the lack of homogeneous questions among the four waves, hindering us from using other relevant variables such as the urban/rural habitat, the parents’ educational level, or the consumption of drugs. Also, available data prevented us from using a detailed measure of binge drinking. Lastly, other proxies of the economic crisis could have been used, and some effects of the economic crisis on health may not be observable in the short term but in a longer period of time.

Despite these limitations, this study presents a new effort to better capture the effect of the economic crisis on adolescents’ health, life satisfaction, and risk behaviors, pointing out the importance of the family socioeconomic position and parental/peer relationships. Childhood is of particular interest for public policies, because of its special vulnerability and because the consequences of childhood deprivation may be perdurable throughout life [52]. The effects of the crises depend to a large extent on social protection policies, on the safety net of the Welfare State, and on the structuring of social and family networks [1].

Therefore, some policy implications derived from this work are related to the importance of maintaining strong active employment policies, as well as social policies focused on single-parent families and less affluent homes. Also, attention should be paid to reducing inequalities between schools, which can be a focus of poor health and unhealthy habits for life. Lastly, given the importance of the child’s social environment, efforts should be made to enhance family communication and support and to avoid bullying.

## 5. Conclusions

Economic crises may have devastated aggregate and individual effects on the population health and wellbeing. Our results confirm that the increase in unemployment experienced in Spain during the recession may have worsened adolescents’ health status and lifestyles. However, the child’s social network seems to play an important protective role against the effects of the economic crisis. The conclusions derived from the work may be relevant to provide more evidence on the importance of designing and implementing public policies that try to alleviate the negative consequences on the well-being of present and future populations, preventing social inequalities and social exclusion. In the future, it would be desirable to make further progress in understanding the short-, medium-, and long-term health consequences of this recent economic crisis on the young population. Also, it may be desirable to examine the relationship between the school context and wider community processes.

## Figures and Tables

**Table 1:** Variables included in the analyses.

Area	Variables	Coding of the Variables
**Dependent Variables**		
**Health Outcomes**	Self-rated health	1 if excellent or good, 0 otherwise.
	Vital satisfaction scale	Continuous variable between 0 and 10, 10 being the best possible life
	Health complaints: 8 physical and psychological symptoms (headache, abdominal pain, backache, feeling dizzy, feeling low, irritable or nervous, and difficulties in getting to sleep)	1 if rarely or never affected by any of them, 0 otherwise
**Risk Behaviors (Lifestyle Habits)**	Smoking	1 if frequently or occasionally smoking during the last year, 0 otherwise
	Alcohol consumption	1 if drinking any alcoholic beverage at least every week (beer, wine, spirits, alcopops, or any other drink that contains alcohol), 0 otherwise
	Absence of breakfast	1 if not eating breakfast every weekday, 0 otherwise
**Control Variables**		
**Individual**	Gender	1 if boy, 0 if girl
	Age groups	Three dummies: 9–12 years, 13–16 years, 17 years and older
**Socioeconomic**	Family structure	1 if biparental, 0 otherwise
	Parents’ work status	1 if both parents are working, 0 otherwise
	Family material wealth (FAS score)	Three dummies: high score, medium score, low score
**Family and Peer Relationships**	Family understanding	1 if the child feels that at least one of the parents understand him/her, 0 otherwise
	Family help	1 if the child feels that at least one of the parents help him/her, 0 otherwise
	Family knowledge	1 if the child feels that at least one of the parents knows a lot about his/her expenditures, friends, leisure time, and night outings, 0 otherwise
	Family love	1 if the child feels that at least one of his/her parents is loving, 0 otherwise
	Physical fights	1 if the child was involved in at least one physical fight with peers during the last year, 0 if otherwise
	Bullying	1 if the child has bullied a peer at least once during the last year, 0 if otherwise
	Bullying victim	1 if the child was bullied by peers at least once during the last year, 0 if otherwise
**Variables Used to Anchor the Multilevel Analysis**		
**Contextual Variables**	School	Dummies for each school: 272 for 2002, 377 for 2006, 133 for 2010, and 399 for 2014
	Region	18 dummies for each Spanish region (17 Autonomous Communities and 1 for Ceuta and Melilla, the two Spanish Autonomous Cities)

**Table 2:** Descriptive analysis of the independent variables.

Category	Description	Year 2002	Year 2006	Year 2010	Year 2014	Total
Sample	Number of children	13,552	21,811	11,230	31,058	77,651
	Number of schools	272	377	133	399	1,181
Gender	% Boys	49.59	48.13	49.39	49.65	49.18
	% Girls	50.41	51.87	50.61	50.35	50.82
Age groups	% Under 13 years	29.85	31.47	27.95	29.98	30.08
	% Aged 13–16 years	53.16	50.35	58.95	59.93	55.92
	% 17 years and older	17.00	18.18	13.10	10.09	14.00
Economic level	% Low family affluence	9.65	5.27	2.99	3.23	5.27
	% Medium family affluence	52.83	45.02	34.33	45.60	44.97
	% High family affluence	37.52	49.71	62.67	51.17	49.76
Parental work status	% Both parents working	62.61	68.88	66.75	62.81	65.08
	% Only one parent working	35.19	29.24	30.53	32.64	31.81
	% No parent working	2.20	1.87	2.72	4.55	3.11
Family structure	% Two-parent	85.92	82.76	79.14	80.23	81.80
	% Single-parent	9.59	10.97	11.46	13.04	11.61
	% Other family structure	4.49	6.27	9.40	6.73	6.59
Family support	% Parental understanding	44.98	69.96	69.90	70.66	65.72
	% Parental help	61.61	87.02	86.10	86.98	82.29
	% Parental knowledge	21.36	47.44	46.05	55.44	43.30
	% Parental love	62.04	87.60	86.13	86.92	82.53
Peer behaviors	% With physical fighting	36.45	31.52	33.17	28.87	22.57
	% Bullying others	31.25	20.11	20.15	17.58	21.33
	% Bullying victims	24.13	12.50	13.33	15.09	15.78
Crisis proxy	Regional unemployment rate annual change (%)	9.12	−7.48	12.07	−7.43	−1.73

**Table 3:** Descriptive analysis of the dependent variables.

Category	Description	Year 2002 (%)	Year 2006 (%)	Year 2010 (%)	Year 2014 (%)	Total (%)
**Health and Life Satisfaction**						
Health symptoms	Rarely/never had health symptoms	51.78	62.31	61.41	61.86	60.12
	Otherwise	48.22	37.69	38.59	38.14	39.88
Self-reported health	Excellent/good	88.37	91.23	91.93	91.85	91.06
	Fair/poor	11.63	8.77	8.07	8.15	8.94
Vital satisfaction Scale (mean scores)		6.57	6.87	6.87	7.70	7.13
**Lifestyles**						
Smoking	Yes	24.92	16.10	17.00	10.00	15.38
	No	75.08	83.90	83.00	90.00	84.62
Frequent alcohol consumption	Yes	17.57	18.29	16.09	7.11	13.42
	No	82.43	81.71	83.91	92.89	86.58
Breakfast during weekdays	5 days	69.24	73.36	61.85	74.61	71.44
	Less than 5 days	30.76	26.64	38.15	25.39	28.56

**Table 4:** Results from the multilevel logistic regressions on health complaints, self-rated health, and life satisfaction (odds ratios/coefficients).

Variable	8 Health Complaints (Logistic)			Self-Rated Health (Logistic)			Life Satisfaction Scale (Linear)		
	Model 1	Model 2	Model 3	Model 1	Model 2	Model 3	Model 1	Model 2	Model 3
Boy	2.196 ***	2.192 ***	2.645 ***	1.630 ***	1.609 ***	1.812 ***	0.146 ***	0.136 ***	0.194 ***
	(0.041)	(0.041)	(0.054)	(0.051)	(0.051)	(0.060)	(0.015)	(0.015)	(0.015)
Aged 13–16	0.590 **	0.594 ***	0.627 ***	0.457 ***	0.465 ***	0.487 ***	−0.776 ***	−0.752 ***	−0.671 ***
	(0.015)	(0.015)	(0.017)	(0.021)	(0.021)	(0.024)	(0.022)	(0.022)	(0.022)
Aged 17 and older	0.451 ***	0.457 ***	0.451 ***	0.334 ***	0.345 ***	0.345 ***	−1.081 ***	−1.040 ***	−0.979 ***
	(0.015)	(0.015)	(0.136)	(0.018)	(0.019)	(0.020)	(0.031)	(0.031)	(0.029)
FAS_medium		1.327 ***	1.220 ***		1.504 ***	1.330 ***		0.400 ***	0.282 ***
		(0.060)	(0.056)		(0.091)	(0.082)		(0.036)	(0.035)
FAS_high		1.471 ***	1.292 ***		1.997 ***	1.640 ***		0.665 ***	0.481 ***
		(0.067)	(0.060)		(0.125)	(0.105)		(0.037)	(0.035)
Both parents working		1.032	1.040 *		1.037	1.040		0.045 ***	0.045 ***
		(0.020)	(0.021)		(0.034)	(0.034)		(0.016)	(0.015)
Two-parent family structure		1.370 ***	1.278 ***		1.444 ***	1.325 ***		0.430 ***	0.337 ***
		(0.037)	(0.036)		(0.060)	(0.056)		(0.022)	(0.021)
Parental understanding			1.269 ***			1.219 ***			0.389 ***
			(0.028)			(0.043)			(0.017)
Parental help			1.218 ***			1.530 ***			0.432 ***
			(0.035)			(0.062)			(0.022)
Parental knowledge			1.479 ***			1.588 ***			0.360 ***
			(0.033)			(0.063)			(0.017)
Parental love			1.242 ***			1.307 ***			0.390 ***
			(0.035)			(0.053)			(0.021)
Fighting with peers			0.642 ***			0.710 ***			−0.192 ***
			(0.015)			(0.026)			(0.017)
Bullying			0.752 ***			0.818 ***			−0.120 ***
			(0.019)			(0.031)			(0.019)
Bullying victim			0.520 ***			0.668 ***			−0.526 ***
			(0.015)			(0.028)			(0.021)
Unemployment rate change	0.470 ***	0.482 ***	1.062	0.548 ***	0.578 ***	1.338	−1.834 ***	−1.769 ***	−0.242 *
	(0.059)	(0.059)	(0.132)	(0.098)	(0.100)	(0.241)	(0.155)	(0.153)	(0.127)
Constant	1.621 ***	0.874 **	0.639 ***	17.296 ***	7.291 ***	4.752 ***	7.608 ***	6.683 ***	5.803 ***
	(0.056)	(0.052)	(0.042)	(0.913)	(0.618)	(0.452)	(0.044)	(0.058)	(0.055)
Observations	53,543	53,543	53,543	56,238	56,238	56,238	55,789	55,789	55,789
Wald chi-squared test	2371	2583	4833	676	928	2009	1754	2742	8997
Likelihood ratio test	383.6	345.6	178.2	146	115	81	2213	2165	1115
MOR/ICC (region)	1.11	1.11	1.08	1.13	1.14	1.13	0.008	0.007	0.004
MOR/ICC (school)	1.32	1.30	1.25	1.38	1.34	1.28	0.077	0.076	0.045

Standard errors in parentheses. *** *p* < 0.01, ** *p* < 0.05, * *p* < 0.1. The models include dummies for the missing values of relationship/conflict indicators. Number of groups: 18 regions; 1,181 schools. Odds ratios and median odds ratios (MOR) for the logistic regression models and coefficients and intraclass correlation coefficients (ICC) for the linear models.

**Table 5:** Results from the multilevel logistic regressions on lifestyle habits (odds ratios).

Variable	Smoking			Alcohol Consumption			Absence of Breakfast		
	Model 1	Model 2	Model 3	Model 1	Model 2	Model 3	Model 1	Model 2	Model 3
Boy	0.751 ***	0.751 ***	0.579 ***	1.517 ***	1.514 ***	1.256 ***	0.627 ***	0.628 ***	0.591 ***
	(0.019)	(0.019)	(0.016)	(0.043)	(0.042)	(0.037)	(0.012)	(0.013)	(0.012)
Aged 13–16	9.585 ***	9.610 ***	8.822 ***	12.048 ***	12.107 ***	10.739 ***	2.132 ***	2.122 ***	1.958 ***
	(0.608)	(0.610)	(0.575)	(1.002)	(1.008)	(0.917)	(0.058)	(0.058)	(0.058)
Aged 17 and older	21.318 ***	21.387 ***	21.982 ***	35.715 ***	36.017 ***	35.044 ***	2.704 ***	2.681 ***	2.490 ***
	(1.467)	(1.475)	(1.566)	(3.139)	(3.169)	(3.177)	(0.096)	(0.095)	(0.095)
FAS_medium		0.894 **	0.943		1.054	1.095		0.780 ***	0.825 ***
		(0.051)	(0.055)		(0.069)	(0.074)		(0.035)	(0.038)
FAS_high		0.844 ***	0.917		1.066	1.137 *		0.722 ***	0.790 ***
		(0.049)	(0.055)		(0.072)	(0.078)		(0.033)	(0.036)
Both parents working		1.077 ***	1.066 **		1.082 ***	1.071 **		0.987	0.983
		(0.029)	(0.030)		(0.032)	(0.032)		(0.020)	(0.021)
Two-parent family structure		0.738 ***	0.801 ***		0.990	1.064		0.736 ***	0.764 ***
		(0.027)	(0.030)		(0.042)	(0.047)		(0.020)	(0.022)
Parental understanding			0.913 ***			0.912 ***			0.840 ***
			(0.027)			(0.029)			(0.019)
Parental help			0.881 ***			0.972			0.849 ***
			(0.031)			(0.039)			(0.024)
Parental knowledge			0.442 ***			0.533 ***			0.753 ***
			(0.014)			(0.018)			(0.018)
Parental love			0.836 ***			0.778 ***			0.913 ***
			(0.029)			(0.030)			(0.026)
Fighting with peers			2.224 ***			1.943 ***			1.226 ***
			(0.067)			(0.064)			(0.029)
Bullying			1.621 ***			1.600 ***			1.128 ***
			(0.051)			(0.055)			(0.029)
Bullying victim			0.752 ***			0.697 ***			1.053 *
			(0.030)			(0.031)			(0.031)
Unemployment rate change	8.493 ***	8.557 ***	2.701 ***	3.424 ***	3.472 ***	0.872	3.660 ***	3.605 ***	2.049 ***
	(2.039)	(2.057)	(0.635)	(1.087)	(1.102)	(0.276)	(0.470)	(0.460)	(0.286)
Constant	0.022 ***	0.032 ***	0.044 ***	0.009 ***	0.008 ***	0.012 ***	0.261 ***	0.452 ***	0.636 ***
	(0.002)	(0.004)	(0.005)	(0.001)	(0.001)	(0.002)	(0.013)	(0.031)	(0.048)
Observations	56,359	56,359	56,359	55,200	55,200	55,200	56,507	56,507	56,507
Wald chi-squared test	2177	2255	4210	2159	2166	3414	1593	1773	2371
Likelihood ratio test	1594	1591	1122	2760	2725	3398	511	504	538
Median odds ratio (region)	1.38	1.39	1.37	1.48	1.48	1.53	1.18	1.18	1.18
Median odds ratio (school)	1.87	1.87	1.76	2.35	2.35	2.24	1.32	1.31	1.33

Standard errors in parentheses. *** *p* < 0.01, ** *p* < 0.05, * *p* < 0.1. The models include dummies for missing values of the categorical contextual variables. Number of groups: 18 regions; 1181 schools.

## Supplementary Materials

The following are available online at https://www.mdpi.com/1660-4601/17/2/643/s1, Table S1: Results from the multilevel logistic regressions on health complaints (odds ratios).

## References

[1] Oliva Moreno J., González López-Valcárcel B., Barber Pérez P., Peña-Longobardo, Urbanos R., Zozaya N. (2018). Crisis Económica y Salud en España.

[2] Parmar D., Stavropoulou C., Ioannidis J.P.A. (2016). Health outcomes during the 2008 financial crisis in Europe: Systematic literature review. BMJ.

[3] Bezruchka S. (2009). The effect of economic recession on population health. CMAJ.

[4] Sawyer S.M., Afifi R.A., Bearinger L.H., Blakemore S.-J., Dick B., Ezeh A.C., Patton G.C. (2012). Adolescence: A foundation for future health. Lancet.

[5] Patel V., Flisher A.J., Hetrick S., McGorry P. (2007). Mental health of young people: A global public-health challenge. Lancet.

[6] Kipping R.R., Campbell R.M., MacArthur G.J., Gunnell D.J., Hickman M. (2012). Multiple risk behaviour in adolescence. J. Public Health (Oxf.).

[7] Kipping R.R., Smith M., Heron J., Hickman M., Campbell R. (2015). Multiple risk behaviour in adolescence and socio-economic status: Findings from a UK birth cohort. Eur. J. Public Health.

[8] Sleskova M., Salonna F., Geckova A.M., Nagyova I., Stewart R.E., van Dijk J.P., Groothoff J.W. (2006). Does parental unemployment affect adolescents’ health?. J. Adolesc. Health.

[9] Moreno Maldonado M.C., Moreno Rodríguez del M.C., Rivera de los Santos F.J. (2016). Indicadores para detectar y evaluar el impacto de las desigualdades socioeconómicas en los estilos de vida y la salud de los adolescentes españoles. Apunt. Psicol..

[10] Padilla-Moledo C., Ruiz J.R., Castro-Piñero J. (2016). Parental educational level and psychological positive health and health complaints in Spanish children and adolescents. Child Care Health Dev..

[11] Zaborskis A., Grincaite M., Lenzi M., Tesler R., Moreno-Maldonado C., Mazur J. (2019). Social Inequality in Adolescent Life Satisfaction: Comparison of Measure Approaches and Correlation with Macro-level Indices in 41 Countries. Soc. Indic. Res..

[12] Gariépy G., Honkaniemi H., Quesnel-Vallée A. (2016). Social support and protection from depression: Systematic review of current findings in Western countries. Br. J. Psychiatry.

[13] Fenton C., Brooks F., Spencer N.H., Morgan A. (2010). Sustaining a positive body image in adolescence: An assets-based analysis. Health Soc. Care Community.

[14] Levin K.A., Currie C. (2010). Adolescent toothbrushing and the home environment: Sociodemographic factors, family relationships and mealtime routines and disorganisation. Community Dent. Oral Epidemiol..

[15] Moreno C., Sánchez-Queija I., Muñoz-Tinoco V., de Matos M.G., Dallago L., Bogt T.T., Camacho I., Rivera F. (2009). HBSC Peer Culture Focus Group Cross-national associations between parent and peer communication and psychological complaints. Int. J. Public Health.

[16] Frasquilho D., de Matos M.G., Marques A., Neville F.G., Gaspar T., Caldas-de-Almeida J.M. (2016). Unemployment, Parental Distress and Youth Emotional Well-Being: The Moderation Roles of Parent–Youth Relationship and Financial Deprivation. Child Psychiatry Hum. Dev..

[17] Pickett W., Simons-Morton B., Dostaler S., Iannotti R.J. (2009). Social environments and physical aggression among 21,107 students in the United States and Canada. J. Sch. Health.

[18] Hale D.R., Patalay P., Fitzgerald-Yau N., Hargreaves D.S., Bond L., Görzig A., Wolpert M., Stansfeld S.A., Viner R.M. (2014). School-Level Variation in Health Outcomes in Adolescence: Analysis of Three Longitudinal Studies in England. Prev. Sci..

[19] West P., Sweeting H., Leyland A. (2004). School effects on pupils’ health behaviours: Evidence in support of the health promoting school. Res. Pap. Educ..

[20] Deschesnes M., Martin C., Hill A.J. (2003). Comprehensive approaches to school health promotion: How to achieve broader implementation?. Health Promot. Int..

[21] Catalano R. (2009). Health, Medical Care, and Economic Crisis. N. Engl. J. Med..

[22] Frasquilho D., de Matos M.G., Gaspar T., Caldas de Almeida J.M. (2016). Young people’s well-being and the economic crisis: How does parental unemployment and family wealth affect the downturn experience?. Child. Youth Serv. Rev..

[23] Frasquilho D., de Matos M.G., Marques A., Gaspar T., Caldas-de-Almeida J.M. (2017). Factors affecting the well-being of adolescents living with unemployed parents in times of economic recession: Findings from the Portuguese HBSC study. Public Health.

[24] Schaller J., Zerpa M. (2019). Short-Run Effects of Parental Job Loss on Child Health. Am. J. Health Econ..

[25] Bacikova-Sleskova M., Benka J., Orosova O. (2015). Parental employment status and adolescents’ health: The role of financial situation, parent-adolescent relationship and adolescents’ resilience. Psychol. Health.

[26] Haisken-DeNew J.P., Kind M. Unexpected Victims: How Parents’ Unemployment Affects Their Children’s Life Satisfaction. https://econpapers.repec.org/paper/iaeiaewps/wp2012n02.htm.

[27] Lundborg P. (2002). Young people and alcohol: An econometric analysis. Addiction.

[28] Ayllón S., Ferreira-Batista N.N. (2018). Unemployment, drugs and attitudes among European youth. J. Health Econ..

[29] Kokkevi A., Stavrou M., Kanavou E. The Repercussions of the Economic Recession in Greece on Adolescents and Their Families. https://www.unicef-irc.org/publications/732-the-repercussions-of-the-economic-recession-in-greece-on-adolescents-and-their-families.html.

[30] Ruhm C.J. (2000). Are Recessions Good for Your Health?. Q. J. Econ..

[31] McClure C.B., Valdimarsdóttir U.A., Hauksdóttir A., Kawachi I. (2012). Economic crisis and smoking behaviour: Prospective cohort study in Iceland. BMJ Open.

[32] Currie C., Nic Gabhainn S., Godeau E., International HBSC Network Coordinating Committee (2009). The Health Behaviour in School-aged Children: WHO Collaborative Cross-National (HBSC) study: Origins, concept, history and development 1982–2008. Int. J. Public Health.

[33] Currie C., Zanotti C., Morgan A., Currie D. (2012). Social Determinants of Health and Well-Being Among Young People, Health Beaviour in School-Aged Children (HBSC) Study: International Report from the 2009/2010 Survey.

[34] Ministerio de Sanidad Consumo y Bienestar Social Estudio HBSC. https://www.mscbs.gob.es/profesionales/saludPublica/prevPromocion/promocion/saludJovenes/estudioHBSC/home.htm.

[35] Tapia Granados J.A. (2005). Increasing mortality during the expansions of the US economy, 1900–1996. Int. J. Epidemiol..

[36] Stuckler D., Basu S., Suhrcke M., Coutts A., McKee M. (2009). The public health effect of economic crises and alternative policy responses in Europe: An empirical analysis. Lancet.

[37] Instituto Nacional de Estadística Encuesta de Población Activa. http://www.ine.es/dynt3/inebase/es/index.htm?padre=982&capsel=985.

[38] Currie C., Molcho M., Boyce W., Holstein B., Torsheim T., Richter M. (2008). Researching health inequalities in adolescents: The development of the Health Behaviour in School-Aged Children (HBSC) family affluence scale. Soc. Sci. Med..

[39] Boyce W., Torsheim T., Currie C., Zambon A. (2006). The Family Affluence Scale as a Measure of National Wealth: Validation of an Adolescent Self-Report Measure. Soc. Indic. Res..

[40] Merlo J., Chaix B., Ohlsson H., Beckman A., Johnell K., Hjerpe P., Råstam L., Larsen K. (2006). A brief conceptual tutorial of multilevel analysis in social epidemiology: Using measures of clustering in multilevel logistic regression to investigate contextual phenomena. J. Epidemiol. Community Health.

[41] Richter M., Vereecken C.A., Boyce W., Maes L., Gabhainn S.N., Currie C.E. (2009). Parental occupation, family affluence and adolescent health behaviour in 28 countries. Int. J. Public Health.

[42] Moore G.F., Cox R., Evans R.E., Hallingberg B., Hawkins J., Littlecott H.J., Long S.J., Murphy S. (2018). School, Peer and Family Relationships and Adolescent Substance Use, Subjective Wellbeing and Mental Health Symptoms in Wales: A Cross Sectional Study. Child Indic. Res..

[43] Karademas E.C., Peppa N., Fotiou A., Kokkevi A. (2008). Family, school and health in children and adolescents: Findings from the 2006 HBSC study in Greece. J. Health Psychol..

[44] Pfoertner T.-K., Rathmann K., Elgar F.J., de Looze M., Hofmann F., Ottova-Jordan V., Ravens-Sieberer U., Bosakova L., Currie C., Richter M. (2014). Adolescents’ psychological health complaints and the economic recession in late 2007: A multilevel study in 31 countries. Eur. J. Public Health.

[45] Ruiz-Pérez I., Bermúdez-Tamayo C., Rodríguez-Barranco M. (2017). Socio-economic factors linked with mental health during the recession: A multilevel analysis. Int. J. Equity Health.

[46] Bond L., Butler H., Thomas L., Carlin J., Glover S., Bowes G., Patton G. (2007). Social and school connectedness in early secondary school as predictors of late teenage substance use, mental health, and academic outcomes. J. Adolesc. Health.

[47] Jose P.E., Ryan N., Pryor J. (2012). Does Social Connectedness Promote a Greater Sense of Well-Being in Adolescence Over Time?. J. Res. Adolesc..

[48] Jiménez-Iglesias A., Camacho I., Rivera F., Moreno C., Matos M.G. (2017). de Social Support from Developmental Contexts and Adolescent Substance Use and Well-Being: A Comparative Study of Spain and Portugal. Span. J. Psychol..

[49] Moreno-Maldonado C., Jiménez-Iglesias A., Rivera F., Moreno C. (2019). Characterization of Resilient Adolescents in the Context of Parental Unemployment. Child Ind. Res..

[50] Morgan A.R., Rivera F., Moreno C., Haglund B.J.A. (2012). Does social capital travel? Influences on the life satisfaction of young people living in England and Spain. BMC Public Health.

[51] Desousa C., Murphy S., Roberts C., Anderson L. (2008). School policies and binge drinking behaviours of school-aged children in Wales—A multilevel analysis. Health Educ. Res..

[52] Oliva J., Peña-Longobardo L.M., López-Valcárcel B.G., Pérez P.B., González N.Z. (2018). Crisis económica y salud: Lecciones aprendidas y recomendaciones para el futuro. Cuad. Económicos ICE.

