# Supplementary material for: The Effect of the Economic Crisis on Adolescents’ Perceived Health and Risk Behaviors: A Multilevel Analysis
Source: Int J Environ Res Public Health. 2020 Jan 19;17(2):643. doi: 10.3390/ijerph17020643 (PMC7013908; doi:10.3390/ijerph17020643)
Supplement: Supplementary file 1 [file ijerph-17-00643-s001.pdf]

Table S1. Results from the multilevel logistic regressions on health complaints (odds ratios).

	**4 physical health complaints**			**4 psychological health complaints**		
	Model 1	Model 2	Model 3	Model 1	Model 2	Model 3
Boy	2.227***	2.226***	2.546***	1.826***	1.821***	2.166***
	(0.043)	(0.043)	(0.052)	(0.033)	(0.033)	(0.042)
Aged 13-16	0.681***	0.684***	0.705***	0.591***	0.596***	0.063***
	(0.017)	(0.017)	(0.019)	(0.014)	(0.014)	(0.016)
Aged 17 and older	0.572***	0.574***	0.560***	0.423***	0.429***	0.427***
	(0.019)	(0.019)	(0.020)	(0.014)	(0.014)	(0.015)
FAS_medium		1.320***	1.245***		1.230***	1.116**
		(0.059)	(0.056)		(0.055)	(0.051)
FAS_high		1.358***	1.240***		1.397***	1.206***
		(0.061)	(0.057)		(0.062)	(0.056)
Both parents working		1.013	1.016		1.044**	1.052***
		(0.020)	(0.021)		(0.020)	(0.021)
Two-parent family structure		1.292***	1.220***		1.336***	1.247***
		(0.035)	(0.034)		(0.036)	(0.034)
Parental understanding			1.168***			1.317***
			(0.026)			(0.028)
Parental help			1.197***			1.231***
			(0.034)			(0.035)
Parental knowledge			1.288***			1.484***
			(0.029)			(0.032)
Parental love			1.126***			1.272***
			(0.032)			(0.036)
Fighting with peers			0.684***			0.643***
			(0.016)			(0.015)
Bullying			0.870***			0.742***
			(0.022)			(0.018)
Bullying victim			0.585***			0.548***
			(0.016)			(0.015)
Unemployment rate change	0.627***	0.639***	1.009	0.432***	0.441***	1.008
	(0.078)	(0.078)	(0.126)	(0.050)	(0.050)	(0.118)
Constant	1.885***	1.133**	0.983	1.237***	0.717***	0.488***
	(0.061)	(0.066)	(0.064)	(0.042)	(0.042)	(0.032)
Observations	54,902	54,902	54,902	54,463	54,463	54,463
Wald chi-squared test	2,055	2,184	3,491	1,919	2,126	4,631
Likelihood Ratio test	320.5	294.9	189.3	315.3	291.3	150.3
Median Odds Ratio (region)	1.09	1.09	1.09	1.11	1.11	1.09
Median Odds Ratio (school)	1.30	1.30	1.25	1.28	1.27	1.22

Standard errors in parentheses. *** p<0.01, ** p<0.05, * p<0.1. The models include dummies for the missing values of relationships/conflicts indicators. No.groups: 18 regions; 1,181 schools.
